# Switching and Discontinuation Pattern of Biologic Disease-Modifying Antirheumatic Drugs and Tofacitinib for Patients With Rheumatoid Arthritis in Taiwan

**DOI:** 10.3389/fphar.2021.628548

**Published:** 2021-07-21

**Authors:** Ko-Jen Li, Chia-Li Chang, Chih-Yi Hsin, Chao-Hsiun Tang

**Affiliations:** ^1^ Department of Internal Medicine, National Taiwan University Hospital, Taipei City, Taiwan; ^2^ School of Health Care Administration, College of Management, Taipei Medical University, Taipei City, Taiwan; ^3^ Pfizer Ltd., Taipei City, Taiwan

**Keywords:** biologic disease-modifying antirheumatic drug, treatment pattern, rheumatoid arthritis, tofacitinib, tumor necrosis factor inhibitors

## Abstract

Rheumatoid arthritis (RA) is a chronic inflammatory systemic disease characterized by persistent joint synovial inflammation and swelling, leading to cartilage damage and bone erosion. This retrospective, longitudinal study is to evaluate the treatment patterns of biologic-naïve RA patients receiving index biologic disease-modifying antirheumatic drug (bDMARD) and tofacitinib by the data of Taiwan National Healthcare Insurance Claims and the Death Registry between 2012 and 2017. Drug survival and treatment patterns were determined by investigating the occurrence of switching and discontinuation from index treatment. At baseline, 70.0% of patients used tumor necrosis factor inhibitors (TNFi) bDMARD with the majority taking etanercept (27.0%) or adalimumab (26.2%). During the follow-up period, 40.0% (*n* = 3,464) of index users switched (*n* = 1,479) or discontinued (*n* = 1,985) the treatment with an average incidence rate of 0.18 per patient-year. Among the six index treatment groups, drug survival was the lowest for adalimumab and highest for tocilizumab. When compared with etanercept, only adalimumab had a higher cumulative probability of switching/discontinuation (adjusted HR = 1.17, 95% CI: 1.08–1.28), whereas golimumab, non-TNFi bDMARDs and tofacitinib were significantly less probable to switch or discontinue. For patients switching the index treatment, tocilizumab (31.2%) and tofacitinib (23.4%) were the main regimens being switched to. In addition, 48.2% of patients who discontinued the index treatment received further retreatment, and 63.8–77.0% of them were retreated with same agent. In conclusion, this population-based study found that TNFi were the preferred agents as the index treatments during 2012–2017. Non-TNFi and tofacitinib were more common second-line agents being switched to. Nearly half of discontinued patients received retreatment, with a majority receiving the same agent.

## Introduction

Rheumatoid arthritis (RA) is a chronic inflammatory systemic disease affecting 0.3–1.0% of the general population with higher prevalence in women and in developed countries (Latimer et al., 2019). The pathophysiology of RA is characterized by persistent joint synovial inflammation and swelling, leading to cartilage damage and bone erosion. Extra-articular manifestations that affect various organs are also common with the progression of RA. Compared with the general population, patients with RA have a higher mortality rate in addition to RA comorbidities, such as cardiovascular, respiratory, and infectious diseases (van den Hoek et al., 2017). The estimated prevalence of RA varied among countries in East Asia (e.g., 0.28% in China, 0.6% in Japan, and 0.32% in South Korea) (Li et al., 2012; Yamanaka et al., 2014; Won et al., 2018). In Taiwan, a nationwide population study using catastrophic illness registry data reported a relatively lower yet rising prevalence of RA from 0.07% in 2002 to 0.12% in 2007 (Kuo et al., 2013).

Management of RA aims to improve the quality of life (QoL) of RA patients by slowing down the progression of joint damage and alleviating the symptoms of the disease. Most guidelines generally recommended conventional synthetic disease-modifying antirheumatic drugs (csDMARDs) as the first-line treatment, followed by switching to or adding biologic DMARDs (bDMARDs) or targeted synthetic DMARDs (tsDMARDs) later in the treatment algorithms when patients demonstrate intolerance or inadequate response to csDMARDs (Lau et al., 2015; Singh et al., 2015; Smolen et al., 2017). In accordance with Taiwan’s National Health Insurance (NHI) drug reimbursement policies, RA patients must have two consecutively disease activity score (DAS) 28 scores >5.1, measured at least one month apart, and have had failure to at least two csDMARDs (including methotrexate [MTX]) to be considered for the reimbursement of bDMARDs and tsDMARDs treatments (Bureau of National Health, 2013).

To date, the approved and reimbursable bDMARDs in Taiwan include tumor necrosis factor inhibitors (TNFi, e.g., adalimumab, etanercept, golimumab, certolizumab and opinercept) and non-TNFi (i.e., bDMARDs with a different mechanism of action [MOA], such as abatacept, a T-cell costimulation inhibitor; tocilizumab, an interleukin-6 receptor inhibitor; or rituximab, an B cell depletion agent). Despite the proven effectiveness of bDMARDs, estimations reveal that approximately 30% of patients may not respond adequately to TNFi and require switching to other bDMARDs (Furst and Emery, 2014). The introduction of tsDMARDs provides an innovative approach to the treatment of RA. Tofacitinib is the first oral Janus kinase (JAK) inhibitor that has been shown to have greater efficacy than csDMARDs (Kremer et al., 2013), and comparable efficacy and safety profile to bDMARDs in moderate-to-severe RA patients (van Vollenhoven et al., 2012; Lee and Bae, 2016; Nakamura et al., 2018). In Taiwan, intra-class bDMARD switching reimbursement is allowed only for patients who demonstrate an adequate response to their current bDMARD treatment but are intolerant to its side effects or wish to reduce the administration frequency of their current agent. Switching to a bDMARD with an alternative mode of action or to tofacitinib is indicated in the NHI policies when patients exhibit poor or loss of treatment response to their current medication (Bureau of National Health, 2013).

The initiation and usage patterns of csDMARDs and bDMARDs in RA patients with inadequate response to MTX with different demographic and patient characteristics have been documented in a number of studies (Ng et al., 2013; Katada et al., 2015; Pope et al., 2018; Olsen et al., 2019). In recent years, the usage pattern, patient adherence, and persistence to tofacitinib in clinical practice have emerged from real-world studies (Caporali and Zavaglia, 2019). Nevertheless, a regional variation on the usage pattern of bDMARDs and tofacitinib in Asia, such as Japan, may be present due to the nature of individual healthcare systems, economic considerations, patient characteristics, and risk factors as compared to Western countries (Harnett et al., 2016; Sugiyama et al., 2016; Bonafede et al., 2018). Therefore, a large scale, population-based cohort study can help us to understand the treatment changes in RA and fill the literature gap among the Asian population. By using the population-based National Health Insurance Research database (NHIRD) in Taiwan, the present study aims to assess the cumulative probability of switching or discontinuation of the index treatment of bDMARDs and tofacitinib, and to describe treatment patterns for patients who switched or were re-treated after discontinuing their index treatment.

## Materials and Methods

### Data Source

This was a retrospective cohort study using the Taiwan population-based claims NHIRD provided by the Taiwan National Health Insurance Administration and maintained by the Health and Welfare Data Science Center (HWDC), Ministry of Health and Welfare, Executive Yuan, Taiwan. The NHIRD captures data from approximately 99% of the Taiwanese population and offers comprehensive patient and clinical information including demographics, diagnosis codes, dates and types of procedures, dispensed prescription drugs, and expenditures (Lin et al., 2018). Death and date of death were identified in the linked Death Registry. All personally identifiable information was encrypted to protect patient privacy. The study was granted an exemption from the Ethical Review Board of the National Taiwan University Hospital.

### Study Patients

**FIGURE 1:**
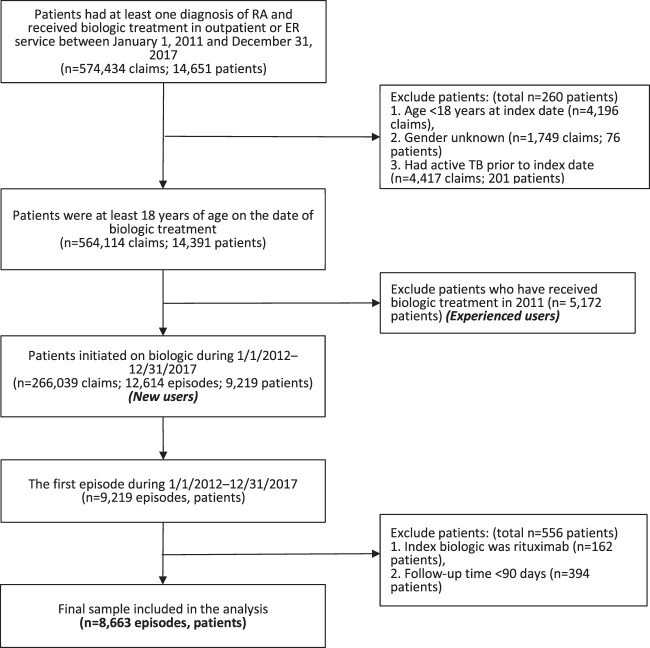
Flow chart of patient selection. Abbreviation: ER, emergency room; RA, rheumatoid arthritis; TB, tuberculosis.

Patients who were at least 18 years of age and had primary or secondary diagnosis of RA (International Classification of Diseases, Ninth Revision, Clinical Modification [ICD-9-CM] codes 714.0 or ICD-10-CM codes M05.7–M05.9, M06.0, M06.2, M06.3, M06.8, M06.9), and received initial treatment of bDMARDs or tofacitinib from January 1, 2012 to December 31, 2017 were eligible for enrollment in the study (*n* = 9,219). We excluded a total of 556 patients who received rituximab as index biologic (*n* = 162) and were followed-up for less than 90 days (*n* = 394). Finally, the study population consisted of 8,663 individuals (Figure 1).

The index date was defined as the date of the first bDMARDs or tofacitinib prescription in NHIRD during the enrollment period. The study patients were then stratified into six groups according to their index treatment: etanercept, adalimumab, golimumab, tocilizumab, abatacept, and tofacitinib.

### Treatment Patterns

The study patients were followed up until the event of switching or discontinuation of index treatment, death, or the end of the data (December 31, 2017), whichever came first. An event of switching occurred when a patient switched to another treatment within a grace period of 90 days after the date of the previous biologics prescription plus drug supply days (see Supplementary Table S1). Discontinuation was defined as patients discontinued the index treatment and had a prescription gap of 90 consecutive days or more (grace period) after the date of the previous prescription plus drug supply days. Patients who did not have any event of switching or discontinuation until death or end of data were considered as continuing the index treatment. Re-treatment was defined as any bDMARD or tofacitinib re-initiated after a gap of 90 days or more.

The Charlson Comorbidity Index (CCI) was used to evaluate comorbidities. Comorbidities were defined as any hospitalization or at least three outpatient claims with primary or secondary diagnosis of the following diseases within one year prior to the index date: chronic obstructive pulmonary disease (COPD; ICD-9-CM codes 490–492,494, 496), hepatitis B (HBV; ICD-9-CM codes V02.61, 070.2, 070.3), hepatitis C (HCV; ICD-9-CM codes V02.62, 070.41, 070.44, 070.51, 070.54, 070.7), diabetes mellitus (DM, ICD-9-CM codes 250), and chronic kidney disease (CKD, ICD-9-CM codes 585).

### Statistical Analysis

Categorical variables were summarized using frequency and percentage, and continuous variables were expressed using mean and standard deviation (SD). The differences in continuous variables and categorical variables among groups were assessed by analysis of variance (ANOVA) and chi-squared test, respectively. Incidence rates of switching/discontinuation were calculated by dividing the number of events by patient-year at risk. The Kaplan-Meier method and the log-rank test were used to examine the difference in drug survival for a maximum follow-up period of 6 years. The Cox proportional-hazards regression was performed to explore the association between index treatment and the cumulative probability of switching/discontinuation, adjusted by covariates which include gender, age, and comorbidities. Use of conventional synthetic DMARDs (csDMARDs) as concomitant medications, such as methotrexate, sulfasalazine, hydroxychloroquine, or leflunomide, as well as corticosteroids were considered as covariates in the regression model. All statistical significances were set at *p* < 0.05, and all analyses were performed using SAS Version 9.4 (Cary, NC, United States) and STATA Version 15.0 (Stata Corp LP, College Station, TX, United States).

## Results

### Baseline Characteristics of Study Patients

**FIGURE 2:**
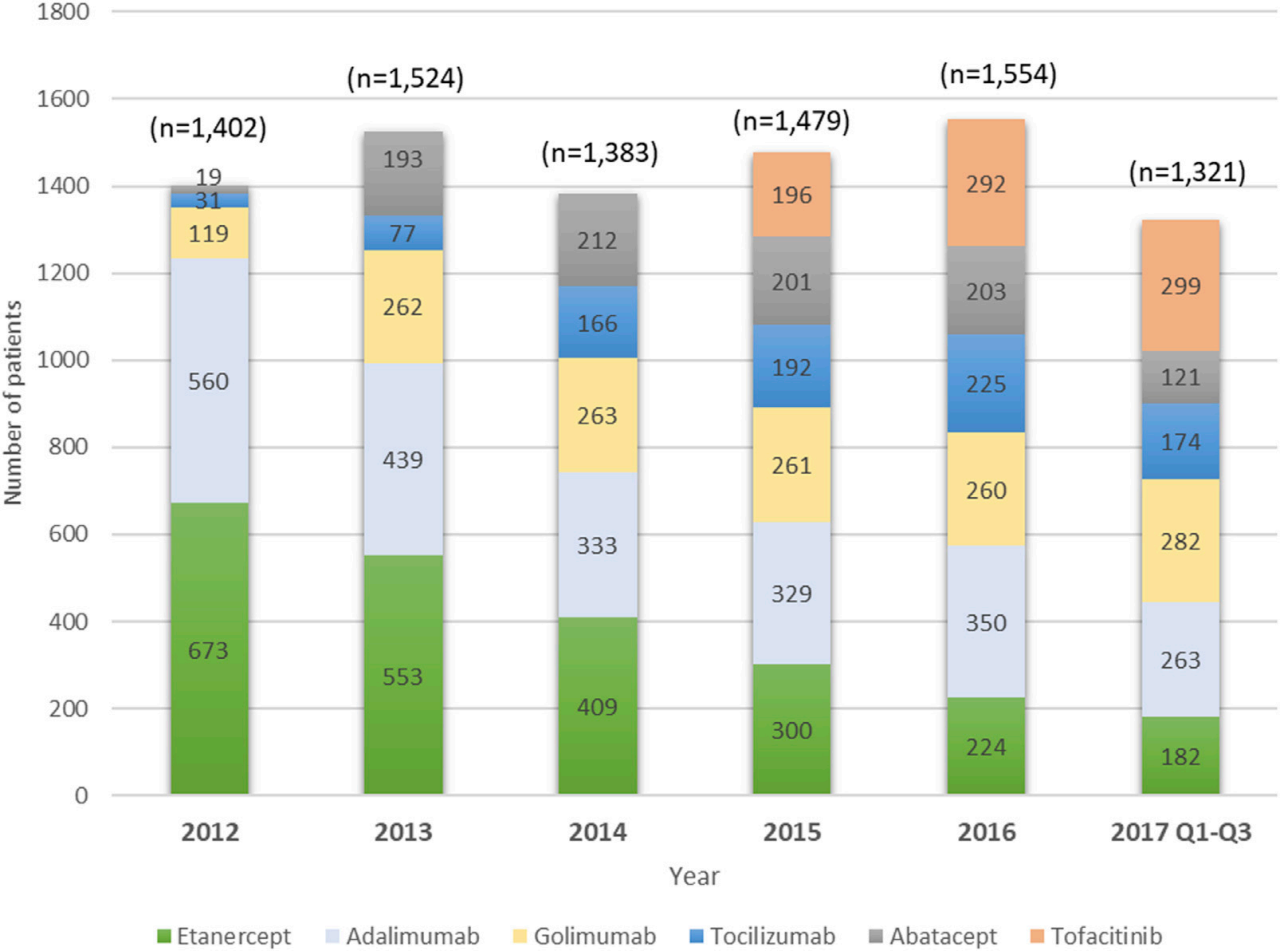
Trend of index treatment, 2012–2017.

A total of 8,663 users of index biologics or tofacitinib with RA diagnosis from January 1, 2012 to September 31, 2017 were included. Table 1 compares the baseline characteristics of patients treated with etanercept, adalimumab, golimumab, tocilizumab, abatacept, and tofacitinib. More than half of the patients were etanercept (27.0%) and adalimumab (26.2%) users. The mean age at diagnosis was 55.1 years (SD 13.5 years), 54.8% of patients were aged 50–69 years old, and 78.3% were women. About 86.3% of patients had been prescribed MTX during one year prior to the index date with mean ± sd of the daily dose (mg) being 1.9 ± 0.4. Age, gender, CCI, HBV, HCV and CKD were significantly different among the six groups. DM (9.9%) and HBV (3.1%) were the most prevalent comorbidities. The proportion of TNFi users, particularly etanercept users, decreased substantially from 2012 to 2017. By contrast, users of non-TNFi (tocilizumab and abatacept) and tofacitinib increased year by year (Figure 2).

**TABLE 1:** Baseline characteristics and treatment patterns of patients by index treatment.

Variable	Total	Index treatment						*p* Value
		Etanercept	Adalimumab	Golimumab	Tocilizumab	Abatacept	Tofacitinib	
	n %(n/N)	n1 %(n1/N1)	n2 %(n2/N2)	n3 %(n3/N3)	n4 %(n4/N4)	n5 %(n5/N5)	n6 %(n6/N6)	
**Number of patients**	(N = 8,663)	(N1 = 2,341)	(N2 = 2,274)	(N3 = 1,447)	(N4 = 865)	(N5 = 949)	(N6 = 787)	
(%)	(100.0%)	(27.0%)	(26.2%)	(16.7%)	(10.0%)	(11.0%)	(9.1%)	
**Index year, n (%)**								
2012	1,402(16.2%)	673(28.7%)	560(24.6%)	119(8.2%)	31(3.6%)	19(2.0%)	0	<0.0001
2013	1,524(17.6%)	553(23.6%)	439(19.3%)	262(18.1%)	77(8.9%)	193(20.3%)	0	
2014	1,383(16.0%)	409(17.5%)	333(14.6%)	263(18.2%)	166(19.2%)	212(22.3%)	0	
2015	1,479(17.1%)	300(12.8%)	329(14.5%)	261(18.0%)	192(22.2%)	201(21.2%)	196(24.9%)	
2016	1,554(17.9%)	224(9.6%)	350(15.4%)	260(18.0%)	225(26.0%)	203(21.4%)	292(37.1%)	
2017	1,321(15.2%)	182(7.8%)	263(11.6%)	282(19.5%)	174(20.1%)	121(12.8%)	299(38.0%)	
**Age at index, mean ± SD**	55.1 ± 13.5	54.3 ± 13.7	53.8 ± 13.6	55.0 ± 13.0	56.1 ± 13.6	57.9 ± 13.3	56.8 ± 13.3	<0.0001
**Age group, n (%)**								
18–29 years	377(4.4%)	129(5.5%)	126(5.5%)	49(3.4%)	35(4.0%)	19(2.0%)	19(2.4%)	<0.0001
30–39 years	809(9.3%)	209(8.9%)	231(10.2%)	137(9.5%)	81(9.4%)	77(8.1%)	74(9.4%)	
40–49 years	1,490(17.2%)	423(18.1%)	395(17.4%)	271(18.7%)	127(14.7%)	145(15.3%)	129(16.4%)	
50–59 years	2,603(30.0%)	717(30.6%)	731(32.1%)	445(30.8%)	254(29.4%)	253(26.7%)	203(25.8%)	
60–69 years	2,146(24.8%)	570(24.3%)	514(22.6%)	338(23.4%)	234(27.1%)	261(27.5%)	229(29.1%)	
≥70 years	1,238(14.3%)	293(12.5%)	277(12.2%)	207(14.3%)	134(15.5%)	194(20.4%)	133(16.9%)	
**Gender, n (%)**								
Female	6,780(78.3%)	1789(76.4%)	1755(77.2%)	1,145(79.1%)	684(79.1%)	767(80.8%)	640(81.3%)	0.0095
Male	1883(21.7%)	552(23.6%)	519(22.8%)	302(20.9%)	181(20.9%)	182(19.2%)	147(18.7%)	
**CCI, mean ± SD**	1.7 ± 1.2	1.7 ± 1.2	1.7 ± 1.2	1.6 ± 1.1	1.8 ± 1.4	1.8 ± 1.2	1.7 ± 1.3	0.0098
**CCI, n (%)**								
CCI≤1	4,757(54.9%)	1,274(54.4%)	1,235(54.3%)	825(57.0%)	483(55.8%)	500(52.7%)	440(55.9%)	0.0321
CCI = 2	2,257(26.1%)	604(25.8%)	624(27.4%)	388(26.8%)	212(24.5%)	246(25.9%)	183(23.3%)	
CCI≥3	1,649(19.0%)	463(19.8%)	415(18.2%)	234(16.2%)	170(19.7%)	203(21.4%)	164(20.8%)	
**Comorbidities, n (%)**								
DM	856(9.9%)	231(9.9%)	206(9.1%)	140(9.7%)	101(11.7%)	91(9.6%)	87(11.1%)	0.2841
HBV	271(3.1%)	86(3.7%)	61(2.7%)	31(2.1%)	31(3.6%)	42(4.4%)	20(2.5%)	0.0096
HCV	159(1.8%)	61(2.6%)	42(1.8%)	20(1.4%)	11(1.3%)	15(1.6%)	10(1.3%)	0.0272
CKD	154(1.8%)	47(2.0%)	31(1.4%)	17(1.2%)	18(2.1%)	28(3.0%)	13(1.7%)	0.0159
COPD	58(0.7%)	16(0.7%)	11(0.5%)	6(0.4%)	8(0.9%)	10(1.1%)	7(0.9%)	0.2940
**% Of any MTX use** ^**a**^	7,476(86.3%)	1952(83.4%)	1978(87.0%)	1,274(88.1%)	738(85.3%)	837(88.2%)	696(88.4%)	0.4852
**Daily dose (mg/day) among the MTX users** **^a^**
Mean ± SD	1.9 ± 0.4	1.9 ± 0.4	1.9 ± 0.4	1.9 ± 0.4	1.8 ± 0.4	1.9 ± 0.4	1.9 ± 0.4	0.8132
Median (IQR)	2.0 (1.7–2.1)	2.0 (1.7–2.1)	2.0 (1.7–2.1)	2.0 (1.7–2.1)	1.9 (1.7–2.1)	2.0 (1.8–2.1)	2.0 (1.8–2.1)	

Abbreviation: DM, diabetes mellitus; HBV, hepatitis B; HCV, hepatitis C; CCI, Charlson Comorbidity Index; CKD, chronic kidney disease; COPD, chronic obstructive pulmonary disease; SD, standard deviation.

a Within one year prior to the index date.

### Incidence of Switching or Discontinuation

**FIGURE 3:**
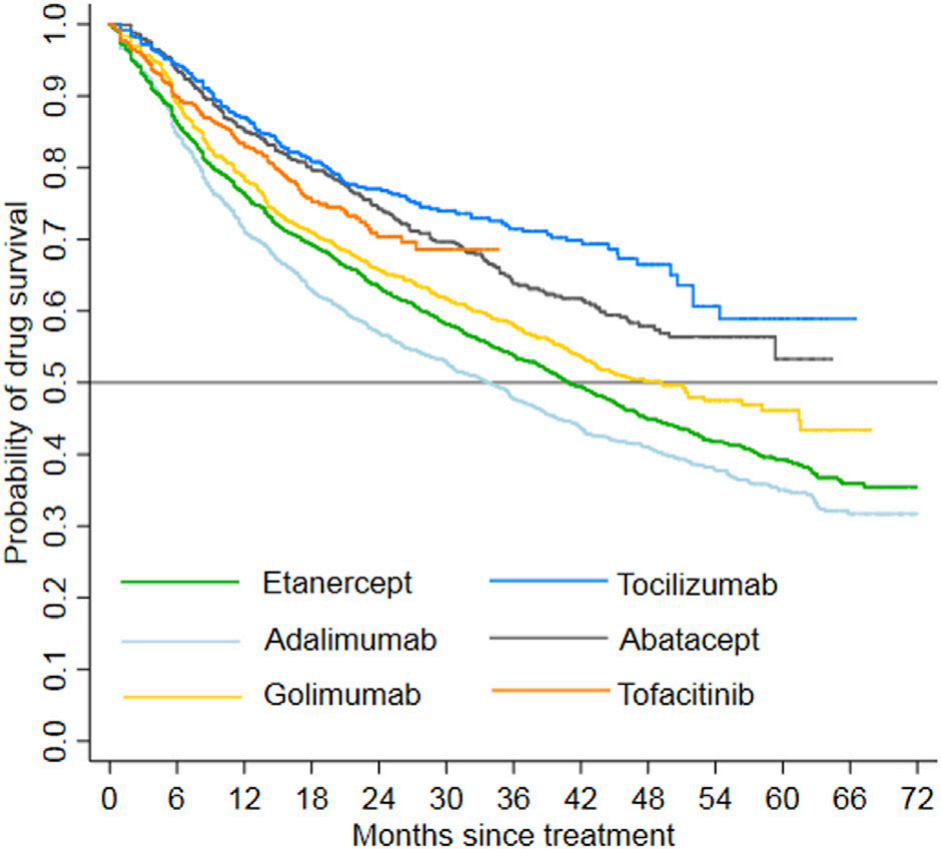
Drug survival by index treatment (Log-rank test, *p* < 0.0001).

The study patients were followed up for a maximum of 6 years and the total follow-up time was 17,531.2 person-years. In total, 1,479 patients switched to other treatment, and 1,985 patients discontinued their index treatment during the follow-up period, respectively. The incidence rate of switching/discontinuation ranged from 1.18 to 2.49 per 10 patient-year among the six index treatment groups, with an average rate of 1.98 per 10 patient-year (Table 2). Kaplan-Meier analyses showed that drug survival was significantly different among the index treatment groups (Figure 3, *p* < 0.0001), with tocilizumab the highest and adalimumab the lowest.

**TABLE 2:** Events and incidence rate of switching or discontinuation.

Variable	Number of patients	Events			Censored			Follow-up time (person-years)	Incidence rate of switching or discontinuation (per 10 person-years)
		Total	Switching	Discontinuation	Total	Death	End of data		
Total	8,663	3,464	1,479	1985	5,199	163	5,036	17,531.2	1.98
Index treatment									
Etanercept	2,341	1,164	474	690	1,177	61	1,116	5,605.3	2.08
Adalimumab	2,274	1,134	457	677	1,140	32	1,108	4,548.9	2.49
Golimumab	1,447	529	260	269	918	16	902	2,799.0	1.89
Tocilizumab	865	193	86	107	672	22	650	1,631.2	1.18
Abatacept	949	287	138	149	662	25	637	2052.2	1.40
Tofacitinib	787	157	64	93	630	7	623	894.6	1.75

### Cox Proportional Regression on Switching/Discontinuation

**FIGURE 4:**
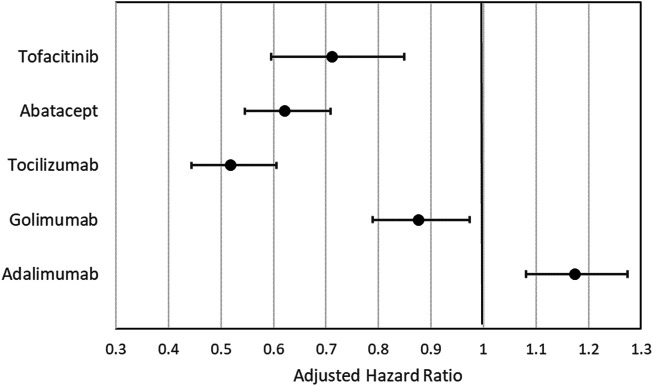
Adjusted hazard ratio (aHR) with 95% confidence interval of the effects of index treatment on the cumulative probability of switching/discontinuation in the Cox proportional regression analysis with etanercept set as the reference group. ^a^Adjusted for age at index, gender; CCI, comorbidities; use of MTX, use of other csDMARDs other than MTX, and use of steroid.

Figure 4 lists the adjusted HRs (aHR) of switching/discontinuation of index treatment in the Cox proportional hazard regression analysis, controlling for index year, age, gender, CCI, comorbidities and use of concomitant medications (see Supplementary Table S2 for complete regression results). When compared with etanercept, adalimumab had a significantly higher probability of switching/discontinuation (aHR = 1.17, 95% CI: 1.08–1.28), whereas golimumab (aHR = 0.88, 95% CI: 0.79–0.97), tocilizumab (aHR = 0.52, 95% CI: 0.44–0.61), abatacept (aHR = 0.62, 95% CI: 0.55–0.71), and tofacitinib (aHR = 0.71, 95% CI: 0.60–0.85) were significantly less probable to switch/discontinuation.

### Treatment Patterns

Among the 1,479 patients who switched to other treatment, non-TNFi were the main agents that patients switched to: tocilizumab (31.2%), tofacitinib (23.4%), abatacept (17.0%), and rituximab (6.6%). Among the 474 patients who switched from index etanercept, 39.2% were switched to tofacitinib (Table 3).

**TABLE 3:** Treatment regimen for patients who switched from the index treatment^a^.

Index treatment	Number of patients who switched	Switching to						
		Etanercept	Adalimumab	Golimumab	Tocilizumab	Abatacept	Tofacitinib	Rituximab
		n1 %(n1/N)	n2 %(n2/N)	n3 %(n3/N)	n4 %(n4/N)	n5 %(n5/N)	n6 %(n6/N)	n7 %(n7/N)
	N							
Total	1,479	75(5.1%)	93(6.3%)	156(10.5%)	461(31.2%)	251(17.0%)	346(23.4%)	97(6.6%)
Etanercept	474	0	58(12.2%)	40(8.4%)	103(21.7%)	63(13.3%)	186(39.2%)	24(5.1%)
Adalimumab	457	27(5.9%)	0	57(12.5%)	184(40.3%)	102(22.3%)	55(12.0%)	32(7.0%)
Golimumab	260	6(2.3%)	13(5.0%)	0	118(45.4%)	58(22.3%)	49(18.8%)	16(6.2%)
Tocilizumab	86	8(9.3%)	9(10.5%)	16(18.6%)	0	23(26.7%)	22(25.6%)	8(9.3%)
Abatacept	138	12(8.7%)	10(7.2%)	23(16.7%)	44(31.9%)	0	34(24.6%)	15(10.9%)
Tofacitinib	64	22(34.4%)	3(4.7%)	20(31.3%)	12(18.8%)	7^a^ (10.9%)		

Abbreviation: HWDC, Health and Welfare Data Science Center.

a Some of the values in the cell were 1 or 2 and thus were combined by HWDC.

Among the 1,985 patients who discontinued their index treatment, 51.8% received no further treatment by the end of the follow-up. Among the 957 patients who were retreated after discontinuation, 63.8–77.0% of them were retreated with the same index treatment (Table 4).

**TABLE 4:** Treatment regimen for patients who discontinued the index treatment.

Index treatment	Number of patients who discontinued	Re-treatment								
		Sub-total	Etanercept	Adalimumab	Golimumab	Tocilizumab	Abatacept	Tofacitinib	No-treatment	
	N	n %(n/N)	n1 %(n1/n)	n2 %(n2/n)	n3 %(n3/n)	n4 %(n4/n)	n5 %(n5/n)	n6 %(n6/n)	n7 %(n7/n)	n8 %(n8/N)
Total	1985	957 (48.2%)	265 (27.7%)	246 (25.7%)	126 (13.2%)	90 (9.4%)	86 (9.0%)	96 (10.0%)	48 (5.0%)	1,028 (51.8%)
Etanercept	690	369 (18.6%)	24 (67.2%)	10 (2.7%)	12 (3.3%)	22 (6.0%)	20 (5.4%)	34 (9.2%)	23 (6.2%)	321 (16.2%)
Adalimumab	677	323 (16.3%)	8 (2.5%)	229 (70.9%)	13 (4.0%)	26 (8.0%)	19 (5.9%)	14 (4.3%)	14 (4.3%)	354 (17.8%)
Golimumab	269	122 (6.1%)	-	3 (2.5%)	94 (77.0%)	9 (7.4%)	4 (3.3%)	7 (5.7%)	3 (2.5%)	147 (7.4%)
Tocilizumab	107	47 (6.8%)	-	-	-	30 (63.8%)	6 (12.8%)	5 (10.6%)	-	60 (3.0%)
Abatacept	149	55 (8.1%)	-	-	-	-	36 (65.5%)	6 (10.9%)	6 (10.9%)	94 (4.7%)
Tofacitinib	93	41 (15.2%)	3 (7.3%)	-	4 (9.8%)	-	-	30 (73.2%)	0	52 (2.6%)

a Some of the values in the cell were covered against some 1 or 2 values being calculated.

## Discussion

In this retrospective cohort study using 2012–2017 population-based claims in the NHIRD, we assessed the initiation and switching/discontinuation patterns in the treatment of biologic-naïve RA patients in Taiwan who used bDMARDs and tofacitinib. This is the first population-based study in Taiwan that investigated the treatment patterns prescribing bDMARDs and tofacitinib from 2012 to 2017, during which several newer classes of biologics were introduced for reimbursement. Due to the comprehensive reimbursement provided by the NHI, the choice of bDMARDs is mostly based on clinical considerations instead of personal financial concerns. Consequently, the inclusion of new agents allows for analysis of the pattern shifts in treatment preference over time in parallel with physicians’ experiences and clinical outcomes.

The current study showed that a majority of patients (70.0%) used TNFi bDMARDs as their index treatment. During the maximum 6-years follow-up study period, adalimumab had a significantly higher cumulative probability of switching/discontinuation, whereas tocilizumab, abatacept, tofacitinib, and golimumab had significantly lower probabilities of switching/discontinuation, in comparison to etanercept. In addition, a majority of switchers (78.1%) used non-TNFi as their next therapy, and almost 77.0% of patients who retreated after discontinuation were retreated with the same index treatment.

Current RA management guidelines do not outline a hierarchal order in which different classes of bDMARDs and tsDMARDs should be used following poor response to csDMARD therapy (Singh et al., 2015; Smolen et al., 2017). On the one hand, the TNFi, etanercept, adalimumab and golimumab, were found in the present study to be the preferred index agents of clinicians to treat the majority of Taiwanese biologic-naïve RA patients during the 6-years observation period. On the other hand, the use of non-TNFi bDMARDs and tofacitinib as index treatment in RA patients is rising in recent years. This observed change of index treatment in Taiwan may reflect that etanercept (in 2003) and adalimumab (in 2004) were reimbursed earlier by NHI than other bDMARDs (in 2012), such as golimumab, tocilizumab and abatacept, as well as tofacitinib (in 2014). The general tendency of prescribing etanercept and adalimumab, over other newly introduced agents was also observed in other countries, such as the United States and Italy (Desai et al., 2017; Silvagni et al., 2018). However, with the availability of newly introduced agents, the distribution of index treatment was affected according to the results of current and previous studies (Kern et al., 2018; Tanaka, 2019).

Drug survival, also known as treatment persistence, is a comprehensive indicator that reflects a combination of treatment effectiveness, safety, and tolerability (Neovius et al., 2015). The current study revealed a lower drug survival rate of TNFi bDMARDs (i.e., adalimumab, etanercept, and golimumab) when compared to non-TNFi bDMARDs (i.e., abatacept and tocilizumab) over the 6-years study period. Recent Japan and US observational studies also reported similar trend (Wei et al., 2017; Ebina et al., 2018). Among TNFi, etanercept showed a higher drug survival rate as compared to adalimumab and infliximab in Europe and US studies (Neovius et al., 2015; Favalli et al., 2016; Souto et al., 2016). When golimumab and non-TNFi were available, treatment persistence was also found to be higher for etanercept than other TNFi in Japan, irrespective of the presence of tofacitinib (Ebina et al., 2018; Kondo and Yamada, 2019). In the present study, the drug survival of adalimumab was the lowest and etanercept did not exhibit a higher survival rate compared to golimumab. This result is probably because a proportion of etanercept index users was switched to tofacitinib.

Switching among biologic therapies should be considered when the first course of treatment exhibits insufficient efficacy or adverse effects (Cannon et al., 2016). Based on several trials and observational studies, clinical management of the first TNFi failure can be conducted by switching to either a second TNFi (cycling strategy) or an alternative targeted agent with a different MOA (swap strategy) (Favalli et al., 2014). Previous studies indicated that switching to new MOA therapy (i.e., abatacept, tocilizumab, or rituximab) or cycling to etanercept was associated with better treatment persistence than cycling to other TNFi (i.e., adalimumab or infliximab) (Favalli et al., 2014; Hirabara et al., 2014; Kobayakawa et al., 2015). According to Taiwan’s NHI reimbursement policies, RA patients who demonstrate a poor response to a biologic treatment should be switched to another biologics with an alternative mode of action or to tofacitinib (Bureau of National Health, 2013). This study found that index users of TNFi preferred to switch to non-TNFi (79.3% for index etanercept and 81.6% for index adalimumab) for further therapy. Tocilizumab and tofacitinib were the most common agents being used for second-line treatment in overall patients who switched from their index therapy. For switching that may be due to adverse effects or clinical conditions, the present study found that, among the patients who switched, 1.5% had diagnosis of pregnancy and 2.7% had been hospitalized due to infections within 3 months before they switched (see Supplementary Table S3).

In addition to switching, 1,028 patients discontinued their index treatment without receiving further treatment by the end of the follow-up. A potential reason for discontinuation in these patients could be related to sustainable remission with effective treatment (Schlager et al., 2019). According to the CORRONA and NinJa collaborative cohort study, roughly 30% of patients with sustained remission discontinued their bDMARD therapy over a 5-year period (Yoshida et al., 2015). Moreover, discontinuation from biologics may occur due to physician preference (Yoshida et al., 2015). Drug withdrawal due to certain clinical conditions (e.g., surgery, pregnancy, or *tuberculosis*) is also a plausible cause; this present study revealed that, among those patients who discontinued their medications, 2.6% of had diagnosis of pregnancy and 7.2% had hospitalization due to infections, including 1.8% with *tuberculosis*, 0.3% with HBV, and 0.2% with herpes zoster, within 3 months before discontinuation (see Supplementary Table S3). Furthermore, 957 patients were retreated after discontinuation, and 63.8–77.0% of these patients were retreated with the same index treatment. However, given the constraint of the observation period in our study, the number of retreatment after discontinuation might be underestimated.

Several caveats must be considered before attempting to apply the findings of this study. First, the major drawback of this study is its observational nature, which may run the risk of confounding biases due to the unmeasured and unknown variables associated with drug survival. Removing such biases may require a randomized control trial. Second, although multivariate regression analysis has been applied to control potential confounders, the independent effects of treatment group on the decision of intra- or inter-biologic switching/discontinuation may be subject to omitted-variable biases, because some important information such as laboratory data, disease duration and disease activity was not available in the dataset. Third, the observation period of this study began in 2012 when both TNFi and non-TNFi biologic agents were listed in the NHI formulary, whereas tofacitinib was only available in NHI after December 1, 2014 (Supplementary Table S1). Compared to other agents, tofacitinib had a shorter follow-up time in this present study; therefore, the drug survival and the switch and discontinuation events of this drug must be assessed further with other agents in a prolonged follow-up period.

In conclusion, based on population-based claims in the NHIRD, this retrospective cohort study found that TNFi were the preferred agents as the index treatments. Non-TNFi (tocilizumab and abatacept) and tofacitinib showed higher drug survival rate compared with etanercept during the 6-years follow-up period. In accordance with Taiwan’s NHI reimbursement policies, non-TNFi bDMARDs and tofacitinib were more common second-line agents being switched to. Among patients who were retreated after discontinuation, the majority of them were retreated with the same index treatment.

## Data Availability

The datasets presented in this article are not readily available because all personally identifiable information was encrypted to protect patient privacy. Requests to access the datasets should be directed to the Health and Welfare Data Science Center (HWDC), Ministry of Health and Welfare, Executive Yuan, Taiwan.

## References

[B1] Bonafede M. M. K. McMorrow D. Proudfoot C. Shinde S. Kuznik A. Chen C. I. (2018). Treatment Persistence and Healthcare Costs Among Patients with Rheumatoid Arthritis after a Change in Targeted Therapy. Am. Health Drug Benefits 11 (4), 192–202. 30464787 30464787 PMC6207310

[B2] Bureau of National Health Insuarance (2013). Provision of the Coverage and Reimbursement Policies/Criteria for Immunologic Agents [Chinese]. Available from: https://www.nhi.gov.tw/Content_List.aspx?n=2A21BF7EBFA32789=5FE8C9FEAE863B46.

[B3] Cannon G. W. DuVall S. L. Haroldsen C. L. Caplan L. Curtis J. R. Michaud K. (2016). Clinical Outcomes and Biologic Costs of Switching between Tumor Necrosis Factor Inhibitors in US Veterans with Rheumatoid Arthritis. Adv. Ther. 33 (8), 1347–1359. 10.1007/s12325-016-0371-0 27352377 PMC4969320

[B4] Caporali R. Zavaglia D. (2019). Real-world Experience with Tofacitinib for the Treatment of Rheumatoid Arthritis. Clin. Exp. Rheumatol. 37 (3), 485–495. 30183607 30183607

[B5] Desai R. J. Solomon D. H. Jin Y. Liu J. Kim S. C. (2017). Temporal Trends in Use of Biologic DMARDs for Rheumatoid Arthritis in the United States: A Cohort Study of Publicly and Privately Insured Patients. Jmcp 23 (8), 809–814. 10.18553/jmcp.2017.23.8.809 28737992 PMC10397716

[B6] Ebina K. Hashimoto M. Yamamoto W. Ohnishi A. Kabata D. Hirano T. (2018). Drug Retention and Discontinuation Reasons between Seven Biologics in Patients with Rheumatoid Arthritis -The ANSWER Cohort Study-. PLoS One 13 (3), e0194130. 10.1371/journal.pone.0194130 29543846 PMC5854351

[B7] Favalli E. G. Biggioggero M. Marchesoni A. Meroni P. L. (2014). Survival on Treatment with Second-Line Biologic Therapy: a Cohort Study Comparing Cycling and Swap Strategies. Rheumatology 53 (9), 1664–1668. 10.1093/rheumatology/keu158 24729445

[B8] Favalli E. G. Pregnolato F. Biggioggero M. Becciolini A. Penatti A. E. Marchesoni A. (2016). Twelve-Year Retention Rate of First-Line Tumor Necrosis Factor Inhibitors in Rheumatoid Arthritis: Real-Life Data from a Local Registry. Arthritis Care Res. 68 (4), 432–439. 10.1002/acr.22788 26556048

[B9] Furst D. E. Emery P. (2014). Rheumatoid Arthritis Pathophysiology: Update on Emerging Cytokine and Cytokine-Associated Cell Targets. Rheumatology 53 (9), 1560–1569. 10.1093/rheumatology/ket414 24402580 PMC4135582

[B10] Harnett J. Gerber R. Gruben D. Koenig A. S. Chen C. (2016). Evaluation of Real-World Experience with Tofacitinib Compared with Adalimumab, Etanercept, and Abatacept in RA Patients with 1 Previous Biologic DMARD: Data from a U.S. Administrative Claims Database. Jmcp 22 (12), 1457–1471. 10.18553/jmcp.2016.22.12.1457 27882833 PMC10397820

[B11] Hirabara S. Takahashi N. Fukaya N. Miyake H. Yabe Y. Kaneko A. (2014). Clinical Efficacy of Abatacept, Tocilizumab, and Etanercept in Japanese Rheumatoid Arthritis Patients with Inadequate Response to Anti-TNF Monoclonal Antibodies. Clin. Rheumatol. 33 (9), 1247–1254. 10.1007/s10067-014-2711-2 24970596 PMC4138439

[B12] Katada H. Yukawa N. Urushihara H. Tanaka S. Mimori T. Kawakami K. (2015). Prescription Patterns and Trends in Anti-rheumatic Drug Use Based on a Large-Scale Claims Database in Japan. Clin. Rheumatol. 34 (5), 949–956. 10.1007/s10067-013-2482-1 24420724

[B13] Kern D. M. Chang L. Sonawane K. Larmore C. J. Boytsov N. N. Quimbo R. A. (2018). Treatment Patterns of Newly Diagnosed Rheumatoid Arthritis Patients from a Commercially Insured Population. Rheumatol. Ther. 5 (2), 355–369. 10.1007/s40744-018-0114-6 29846932 PMC6251837

[B14] Kobayakawa T. Kojima T. Takahashi N. Hayashi M. Yabe Y. Kaneko A. (2015). Drug Retention Rates of Second Biologic Agents after Switching from Tumor Necrosis Factor Inhibitors for Rheumatoid Arthritis in Japanese Patients on Low-Dose Methotrexate or without Methotrexate. Mod. Rheumatol. 25 (2), 251–256. 10.3109/14397595.2014.953668 25211402

[B15] Kondo M. Yamada H. (2019). Drug Survival Rates of Biological Disease-Modifying Antirheumatic Drugs and Janus Kinase-Inhibitor Therapy in 801 Rheumatoid Arthritis Patients: a 14 Year-Retrospective Study from a Rheumatology Clinic in Japan. Mod. Rheumatol. 29, 928–935. 10.1080/14397595.2018.1537556 30334661

[B16] Kremer J. Li Z.-G. Hall S. Fleischmann R. Genovese M. Martin-Mola E. (2013). Tofacitinib in Combination with Nonbiologic Disease-Modifying Antirheumatic Drugs in Patients with Active Rheumatoid Arthritis. Ann. Intern. Med. 159 (4), 253–261. 10.7326/0003-4819-159-4-201308200-00006 24026258

[B17] Kuo C. F. Luo S. F. See L. C. Chou I. J. Chang H. C. Yu K. H. (2013). Rheumatoid Arthritis Prevalence, Incidence, and Mortality Rates: a Nationwide Population Study in Taiwan. Rheumatol. Int. 33 (2), 355–360. 10.1007/s00296-012-2411-7 22451027

[B18] Latimer N. White I. Abrams K. Siebert U. (2019). Causal Inference for Long-Term Survival in Randomised Trials with Treatment Switching: Should Re-censoring Be Applied when Estimating Counterfactual Survival Times? Stat. Methods Med. Res. 28 (8), 2475–2493. 10.1177/0962280218780856 29940824 PMC6676341

[B19] Lau C. S. Chia F. Harrison A. Hsieh T. Y. Jain R. Jung S. M. (2015). APLAR Rheumatoid Arthritis Treatment Recommendations. Int. J. Rheum. Dis. 18 (7), 685–713. 10.1111/1756-185X.12754 26334449

[B20] Lee Y. H. Bae S. C. (2016). Comparative Efficacy and Safety of Tocilizumab, Rituximab, Abatacept and Tofacitinib in Patients with Active Rheumatoid Arthritis that Inadequately Responds to Tumor Necrosis Factor Inhibitors: a Bayesian Network Meta-Analysis of Randomized Controlled Tri. Int. J. Rheum. Dis. 19 (11), 1103–1111. 10.1111/1756-185X.12822 26692536

[B21] Li R. Sun J. Ren L.-M. Wang H. Y. Liu W. H. Zhang X. W. (2012). Epidemiology of Eight Common Rheumatic Diseases in China: a Large-Scale Cross-Sectional Survey in Beijing. Rheumatology 51 (4), 721–729. 10.1093/rheumatology/ker370 22179737

[B22] Lin L.-y. Warren-Gash C. Smeeth L. Chen P.-C. (2018). Data Resource Profile: the National Health Insurance Research Database (NHIRD). Epidemiol. Health 40, e2018062. 10.4178/epih.e2018062 30727703 PMC6367203

[B23] Nakamura Y. Suzuki T. Yamazaki H. Kato H. (2018). Tofacitinib versus Non-tumor Necrosis Factor Biologics for Patients with Active Rheumatoid Arthritis. Arch. Rheumatol. 33 (2), 154–159. 10.5606/ArchRheumatol.2018.6366 30207562 PMC6117139

[B24] Neovius M. Arkema E. V. Olsson H. Eriksson J. K. Kristensen L. E. Simard J. F. (2015). Drug Survival on TNF Inhibitors in Patients with Rheumatoid Arthritis Comparison of Adalimumab, Etanercept and Infliximab. Ann. Rheum. Dis. 74 (2), 354–360. 10.1136/annrheumdis-2013-204128 24285495 PMC4316855

[B25] Ng B. Chu A. Khan M. M. (2013). A Retrospective Cohort Study: 10-year Trend of Disease-Modifying Antirheumatic Drugs and Biological Agents Use in Patients with Rheumatoid Arthritis at Veteran Affairs Medical Centers. BMJ Open 3 (4), e002468. 10.1136/bmjopen-2012-002468 PMC3641511 23562815

[B26] Olsen I. C. Lie E. Vasilescu R. Wallenstein G. Strengholt S. Kvien T. K. (2019). Assessments of the Unmet Need in the Management of Patients with Rheumatoid Arthritis: Analyses from the NOR-DMARD Registry. Rheumatology (Oxford, England) 58 (3), 481–491. 10.1093/rheumatology/key338 PMC6381770 30508189

[B27] Pope J. E. Rampakakis E. Movahedi M. Cesta A. Li X. Couto S. (2018). Treatment Patterns in Rheumatoid Arthritis after Discontinuation of Methotrexate: Data from the Ontario Best Practices Research Initiative (OBRI). Clin. Exp. Rheumatol. 36 (2), 215–222. 29148403

[B28] Schlager L. Loiskandl M. Aletaha D. Radner H. (2019). Predictors of Successful Discontinuation of Biologic and Targeted Synthetic DMARDs in Patients with Rheumatoid Arthritis in Remission or Low Disease Activity: a Systematic Literature Review. Rheumatology, 59.Oxford, England, 324–334. 10.1093/rheumatology/kez278 31325305

[B29] Silvagni E. Bortoluzzi A. Carrara G. Zanetti A. Govoni M. Scirè C. A. (2018). Comparative Effectiveness of First-Line Biological Monotherapy Use in Rheumatoid Arthritis: a Retrospective Analysis of the RECord-Linkage on Rheumatic Diseases Study on Health Care Administrative Databases. BMJ Open 8 (9), e021447. 10.1136/bmjopen-2017-021447 PMC6144331 30206082

[B30] Singh J. A. Saag K. G. Bridges S. L. Jr. Akl E. A. Bannuru R. R. Sullivan M. C. (2015). 2015 American College of Rheumatology Guideline for the Treatment of Rheumatoid Arthritis. Arthritis Rheumatol. 68 (1), 1–26. 10.1002/art.39480 26545940

[B31] Smolen J. S. Landewé R. Bijlsma J. Burmester G. Chatzidionysiou K. Dougados M. (2017). EULAR Recommendations for the Management of Rheumatoid Arthritis with Synthetic and Biological Disease-Modifying Antirheumatic Drugs: 2016 Update. Ann. Rheum. Dis. 76 (6), 960–977. 10.1136/annrheumdis-2016-210715 28264816

[B32] Souto A. Maneiro J. R. Gómez-Reino J. J. (2016). Rate of Discontinuation and Drug Survival of Biologic Therapies in Rheumatoid Arthritis: a Systematic Review and Meta-Analysis of Drug Registries and Health Care Databases. Rheumatology 55 (3), kev374–34. 10.1093/rheumatology/kev374 26490106

[B33] Sugiyama N. Kawahito Y. Fujii T. Atsumi T. Murata T. Morishima Y. (2016). Treatment Patterns, Direct Cost of Biologics, and Direct Medical Costs for Rheumatoid Arthritis Patients: A Real-World Analysis of Nationwide Japanese Claims Data. Clin. Ther. 38 (6), 1359–1375. 10.1016/j.clinthera.2016.03.022 27101816

[B34] Tanaka Y. The JAK inhibitors: Do they bring a paradigm shift for the management of rheumatic diseases? Rheumatology (Oxford, England) (2019) 58, i1, i3. 10.1093/rheumatology/key280 30806705 PMC6390877

[B35] van den Hoek J. Boshuizen H. C. Roorda L. D. Tijhuis G. J. Nurmohamed M. T. van den Bos G. A. M. (2017). Mortality in Patients with Rheumatoid Arthritis: a 15-year Prospective Cohort Study. Rheumatol. Int. 37 (4), 487–493. 10.1007/s00296-016-3638-5 28032180 PMC5357293

[B36] van Vollenhoven R. F. Fleischmann R. Cohen S. Lee E. B. García Meijide J. A. Wagner S. (2012). Tofacitinib or Adalimumab versus Placebo in Rheumatoid Arthritis. N. Engl. J. Med. 367 (6), 508–519. 10.1056/NEJMoa1112072 22873531

[B37] Wei W. Knapp K. Wang L. Chen C.-I. Craig G. L. Ferguson K. (2017). Treatment Persistence and Clinical Outcomes of Tumor Necrosis Factor Inhibitor Cycling or Switching to a New Mechanism of Action Therapy: Real-World Observational Study of Rheumatoid Arthritis Patients in the United States with Prior Tumor Necrosis Factor Inhibitor Therapy. Adv. Ther. 34 (8), 1936–1952. 10.1007/s12325-017-0578-8 28674959 PMC5565674

[B38] Won S. Cho S. K. Kim D. Han M. Lee J. Jang E. J. (2018). Update on the Prevalence and Incidence of Rheumatoid Arthritis in Korea and an Analysis of Medical Care and Drug Utilization. Rheumatol. Int. 38 (4), 649–656. 10.1007/s00296-017-3925-9 29302803

[B39] Yamanaka H. Sugiyama N. Inoue E. Taniguchi A. Momohara S. (2014). Estimates of the Prevalence of and Current Treatment Practices for Rheumatoid Arthritis in Japan Using Reimbursement Data from Health Insurance Societies and the IORRA Cohort (I). Mod. Rheumatol. 24 (1), 33–40. 10.3109/14397595.2013.854059 24261756

[B40] Yoshida K. Radner H. Mjaavatten M. D. Greenberg J. D. Kavanaugh A. Kishimoto M. (2015). Incidence and Predictors of Biological Antirheumatic Drug Discontinuation Attempts Among Patients with Rheumatoid Arthritis in Remission: A CORRONA and NinJa Collaborative Cohort Study. J. Rheumatol. 42 (12), 2238–2246. 10.3899/jrheum.150240 26523025

