# Supplementary material for: Switching and Discontinuation Pattern of Biologic Disease-Modifying Antirheumatic Drugs and Tofacitinib for Patients With Rheumatoid Arthritis in Taiwan
Source: Front Pharmacol. 2021 Jul 21;12:628548. doi: 10.3389/fphar.2021.628548 (PMC8333863; doi:10.3389/fphar.2021.628548)
Supplement: Supplementary file 1 [file DataSheet1.docx]

**Supplementary Material**

**Supplementary Table S1.** The reimbursement date and drug supply days^a^ of bDMARD and tofacitinib

| **Agent** | **Dosage (mg)** | **Drug supply (days)** | **Reimbursement start date in Taiwan** |
| --- | --- | --- | --- |
| Etanercept | 25 | 3.5 | 2003/3/1 |
|  | 50 | 7 | 2013/10/1 |
| Adalimumab | 40 | 14 | 2004/9/1 |
| Rituximab | 100 | 180 | 2008/11/1 |
| Golimumab | 50–100 | 28 | 2012/1/1 |
| Tocilizumab | 80, 200, 400 | 28 | 2012/5/1 |
| Abatacept | 250 | 7 | 2012/6/1 |
|  | 125 | 28 | 2015/8/1 |
| Tofacitinib | 5 | 0.5 | 2014/12/1 |
|  | 11 | 1 | 2018/6/1 |

Abbreviation: bDMARD, biologic disease-modifying antirheumatic drug.

^a^ Drug supply days = Imputed days’ supply.

**Supplementary Table S2.** Cox proportional regression analysis of the cumulative probability of switching/discontinuation (for a maximum follow-up of 6 years)

| Variable | **Univariate analysis** | | | |  | **Multivariate analysis^a^** | | | |
| --- | --- | --- | --- | --- | --- | --- | --- | --- | --- |
|  | cHR | 95% CI | | *P* value |  | aHR | 95% CI | | *P* value |
| **Index treatment** |  |  |  |  |  |  |  |  |  |
| Etanercept | 1.00 | (Ref.) | | |  | 1.00 | (Ref.) | | |
| Adalimumab | 1.16 | 1.07 | 1.26 | 0.0003 |  | 1.17 | 1.08 | 1.28 | 0.0001 |
| Golimumab | 0.87 | 0.78 | 0.96 | 0.0069 |  | 0.88 | 0.79 | 0.97 | 0.0131 |
| Tocilizumab | 0.53 | 0.46 | 0.62 | <.0001 |  | 0.52 | 0.44 | 0.61 | <.0001 |
| Abatacept | 0.65 | 0.57 | 0.73 | <.0001 |  | 0.62 | 0.55 | 0.71 | <.0001 |
| Tofacitinib | 0.71 | 0.6 | 0.84 | <.0001 |  | 0.71 | 0.60 | 0.85 | 0.0002 |
| **Index year** |  |  |  |  |  |  |  |  |  |
| <=2014 |  |  |  |  |  | 1.00 | (Ref.) | | |
| >=2015 |  |  |  |  |  | 1.04 | 0.96 | 1.13 | 0.3750 |
| **Age at index** |  |  |  |  |  |  |  |  |  |
| 18–29 years |  |  |  |  |  | 1.00 | (Ref.) | | |
| 30–39 years |  |  |  |  |  | 0.91 | 0.75 | 1.09 | 0.2956 |
| 40–49 years |  |  |  |  |  | 0.79 | 0.67 | 0.94 | 0.0081 |
| 50–59 years |  |  |  |  |  | 0.85 | 0.72 | 1.00 | 0.0449 |
| 60–69 years |  |  |  |  |  | 0.90 | 0.76 | 1.07 | 0.2274 |
| ≥70 years |  |  |  |  |  | 1.07 | 0.90 | 1.28 | 0.4590 |
| **Gender** |  |  |  |  |  |  |  |  |  |
| Female |  |  |  |  |  | 1.00 | (Ref.) | | |
| male |  |  |  |  |  | 1.01 | 0.93 | 1.10 | 0.8328 |
| **CCI** |  |  |  |  |  |  |  |  |  |
| CCI≤1 |  |  |  |  |  | 1.00 | (Ref.) | | |
| CCI=2 |  |  |  |  |  | 1.02 | 0.94 | 1.10 | 0.7263 |
| CCI≥3 |  |  |  |  |  | 1.03 | 0.93 | 1.14 | 0.6257 |
| **Comorbidities** |  |  |  |  |  |  |  |  |  |
| HBV |  |  |  |  |  | 0.90 | 0.74 | 1.10 | 0.2984 |
| HCV |  |  |  |  |  | 0.95 | 0.74 | 1.21 | 0.6607 |
| CKD |  |  |  |  |  | 1.18 | 0.92 | 1.51 | 0.1931 |
| COPD |  |  |  |  |  | 1.33 | 0.92 | 1.91 | 0.1275 |
| DM |  |  |  |  |  | 1.10 | 0.98 | 1.24 | 0.1125 |
| **Concomitant medications**^b^ |  |  |  |  |  |  |  |  |  |
| Steroid |  |  |  |  |  | 1.35 | 1.21 | 1.51 | <.0001 |
| csDMARD, MTX |  |  |  |  |  | 0.83 | 0.76 | 0.91 | <.0001 |
| csDMARD, Non-MTX^c^ |  |  |  |  |  | 1.18 | 1.05 | 1.34 | 0.0065 |

Abbreviation: aHR, adjusted hazard ratio; CCI, Charlson Comorbidity Index; cHR, crude hazard ratio; CI, confidence interval; CKD, chronic kidney disease; COPD, chronic obstructive pulmonary disease; csDMARD, conventional synthetic disease-modifying antirheumatic drugs; DM, diabetes mellitus; HBV, hepatitis B; HCV, hepatitis C; MTX, methotrexate; Ref., reference group.

^a^Adjusted for age at index, gender, CCI, and comorbidities, use of MTX, use of other csDMARDs other than MTX, and use of steroid.

^b^Concomitant medications is followed from index date to switching/discontinuation or end of data.

^c^Hydroxychloroquine, leflunomide and sulfasalazine

**Supplementary Table S3.** Events occurred within 3 months prior to the date of switch or discontinuation

|  | Among patients who switched or discontinued their index biologic | | | Observation period | Definition of events |
| --- | --- | --- | --- | --- | --- |
|  | **Total** | **Switched** | **Discontinued** |  |  |
|  | **(N=3,464)** | **(N=1,479)** | **(N=1,985)** |  |  |
| Pregnancy | 74 (2.1%) | 22 (1.5%) | 52 (2.6%) | Within 3  months (90  days) prior to  the date of  switched or  discontinuation | Had any claims of prenatal visits |
| Had hospitalization due to infections | 182 (5.3%) | 40 (2.7%) | 142 (7.2%) |  | Had principal/secondary diagnosis of infections. ((https://www.questdiagnostics.com/dms/Documents/Other/CPT-  2015/ICD_9-10_Infectious_Disease_MI4956.pdf) |
| Tuberculosis | 43 (1.2%) | 7 (0.5%) | 36 (1.8%) |  | ICD-9 codes 010-018, 137.0 or ICD-10 codes A15.7, A15.0, A15.8, A17.0, A18.01, A18.02, A18.03, A18.09, A18.4, A19.9, B90.9 |
| HBV | 6 (0.2%) | 1 (0.1%) | 5 (0.3%) |  | ICD-9 codes 070.32 or ICD-10 codes B18.1 |
| Herpes zoster | 3 (0.1%) | 0 (0%) | 3 (0.2%) |  | ICD-9 codes 053.9 or ICD-10 codes B02.9 |
